# Estimation of Mechanical Power Output Employing Deep Learning on Inertial Measurement Data in Roller Ski Skating

**DOI:** 10.3390/s21196500

**Published:** 2021-09-29

**Authors:** Md Zia Uddin, Trine M. Seeberg, Jan Kocbach, Anders E. Liverud, Victor Gonzalez, Øyvind Sandbakk, Frédéric Meyer

**Affiliations:** 1 SINTEF Digital, 0373 Oslo, Norway; zia.uddin@sintef.no (M.Z.U.); Trine.Seeberg@sintef.no (T.M.S.); anders.liverud@sintef.no (A.E.L.); victor.gonzalez@sintef.no (V.G.); 2 Centre for Elite Sports Research, Department of Neuromedicine and Movement Science, Norwegian University of Science and Technology, 7491 Trondheim, Norway; jan.kocbach@gmail.com (J.K.); oyvind.sandbakk@ntnu.no (Ø.S.); 3 Department of Informatics, University of Oslo, 0316 Oslo, Norway

**Keywords:** cross country skiing, IMU, wearable sensors, LSTM, neural network

## Abstract

The ability to optimize power generation in sports is imperative, both for understanding and balancing training load correctly, and for optimizing competition performance. In this paper, we aim to estimate mechanical power output by employing a time-sequential information-based deep Long Short-Term Memory (LSTM) neural network from multiple inertial measurement units (IMUs). Thirteen athletes conducted roller ski skating trials on a treadmill with varying incline and speed. The acceleration and gyroscope data collected with the IMUs were run through statistical feature processing, before being used by the deep learning model to estimate power output. The model was thereafter used for prediction of power from test data using two approaches. First, a user-dependent case was explored, reaching a power estimation within 3.5% error. Second, a user-independent case was developed, reaching an error of 11.6% for the power estimation. Finally, the LSTM model was compared to two other machine learning models and was found to be superior. In conclusion, the user-dependent model allows for precise estimation of roller skiing power output after training the model on data from each athlete. The user-independent model provides less accurate estimation; however, the accuracy may be sufficient for providing valuable information for recreational skiers.

## 1. Introduction

Cross-country (XC) and roller skiing are endurance sports performed in varying terrain with subsequent variations in speed, as well as both external and metabolic power [1,2,3]. The varying terrain of the courses used during training and competition induces periods of very high intensity during uphill stints, and the ability to recover in downhill stints [4]. In addition, terrain, track and weather conditions influence the opposing forces and constraints for producing power through poles and skis, which have a high impact on skiing speed at a given metabolic intensity [5]. It is, therefore, not feasible to compare the performance of athletes from day-to-day or from track-to-track by using speed or segment time, as in many other sports, such as running or cycling. This highlights a need for metrics, which can be used to track and compare performance, independently of track, terrain and weather conditions.

Power meters, measuring the power output defined as the product of force and velocity, are used extensively in cycling to quantitatively track changes in fitness and performance [6]. However, while mechanical power can be directly measured on the bike with force sensors (on the pedals, in the pedal or in the wheel), measurement of power output in XC skiing is more complex, as force magnitude and direction must be measured for both poles and skis [7]. Therefore, power output is commonly estimated using a power balance model, but the accuracy of these estimations have so far been too low for most practical applications [8,9]. In running, the direct measurement of power output is also challenging, and thus an indirect approach, where the mechanical power is estimated using inertial measurement units (IMUs), has been attempted with several commercial technologies on the market. However, the proprietary methods used for power estimation in running from IMU data are not published, and the repeatability and concurrent validity of the commercial technologies is found to be low [10,11,12].

In the past decade, wearable devices, such as IMUs, have encountered success in sport science applications, as they allow for field analysis thanks to their high accuracy, small size and light mass. In XC skiing, IMUs have been used to determine spatio-temporal parameters [13] and to classify the used sub-techniques [14,15,16]. Machine learning has also been used to perform sub-technique classification [17]. As the power determination in XC and roller skiing relies on complex mechanisms [9], a machine-learning-based approach using multiple IMUs on the athlete could therefore be a relevant method to estimate power output. 

To model and decode time-sequential information in input sensor data, deep learning models are promising [18]. Among all the different deep models, the Deep Belief Network (DBN) was the first to be successful, as it was faster than the typical large artificial neural network [19]. To improve upon that, Convolutional Neural Networks (CNNs) were proposed, mostly for visual pattern recognition, which had the ability of creating important features while traversing the nodes of the machine learning models [20]. Though CNN has better data modelling power than DBN, it seems that the typical CNNs are not suitable for time-sequential modelling of data. Recurrent Neural Networks (RNNs) have the advantage over the aforementioned other models (e.g., DBN and CNN), which model time-sequential data obtained from different sources, such as sensors [21,22,23,24,25,26,27]. However, typical RNNs have the limitation of vanishing gradient problems while modelling long sequences of data. This problem has been overcome by the Long Short-Term Memory (LSTM) method, which basically introduces different memory units over typical RNN mechanism. Hence, this work adopts LSTM to develop machine learning models for ski-power estimation.

The aim of this paper was to estimate mechanical power output during roller ski skating, using an LSTM machine learning method on IMU sensor data, and assess the accuracy of the developed algorithms. We made the hypothesis that models including individualized information would give a good accuracy, allowing for inter- and intra-subject comparisons, while user-independent models would reach average accuracy, allowing only for overall behaviour analysis.

## 2. Material and Methods

### 2.1. Participants

Thirteen elite male Norwegian skiers, consisting of eight XC skiers (distance FIS points: 47 ± 21) and five biathletes, participated in the study (age 24.8 ± 2.7 years; body height 184 ± 6 cm; body mass 79.3 ± 5.2 kg, VO_2max_ 69.5 ± 3.6 mL·min^−1^·kg^−1^). All skiers were healthy and free of injuries at the time of testing and were accustomed to treadmill roller skiing. More details about the data collection and participants are given in Seeberg et al. [28].

### 2.2. Equipment

**Figure 1:**
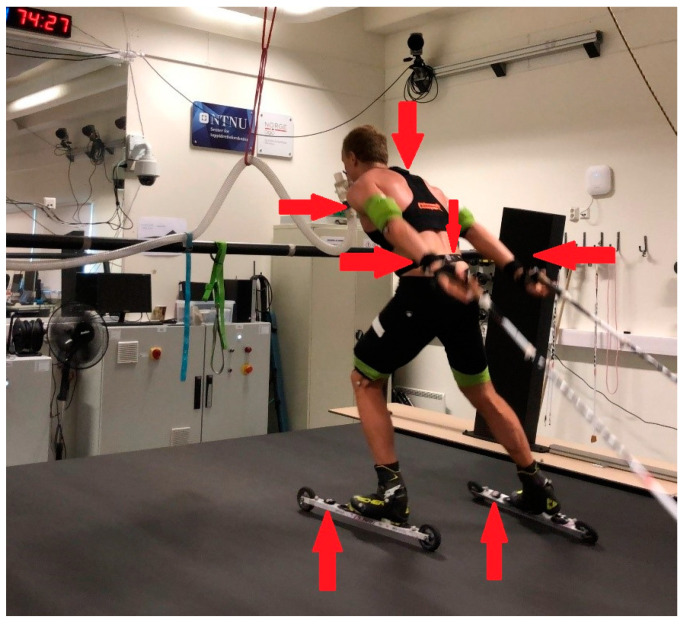
Overview of the experimental setup with an athlete skiing on the treadmill. Red arrows show the position of the sensors.

The protocol was performed on a 3-by-5 m motor-driven treadmill on roller skis (Forcelink S-mill, Motekforce Link, Amsterdam, The Netherlands) (Figure 1). The skiers used poles of their individually chosen lengths with special carbide tips. All skiers wore their own skating XC shoes but used the same pair of Skate Elite roller skis (IDT Sports, Lena, Norway) with an NNN binding system (Rottefella, Klokkarstua, Norway) and with standard category 2 wheels to minimize variations in roller resistance. The rolling friction coefficient (μ) was tested before, at various times during, and after the study, using a towing test [29], providing an average μ-value of 0.016. The skiers wore a safety harness connected to an automatic emergency brake at the high-intensity parts of the tests. Incline and speed of the treadmill were calibrated before and after the study by using the Qualisys Pro Reflex system and Qualisys Track Manager software (Qualisys AB, Gothenburg, Sweden).

Before testing, the body mass of each skier was determined with an electronic body-mass scale (Seca model nr:877; Seca GmbH & Co. KG., Hamburg, Germany). Movement data were collected by seven IMUs, one Optimeye S5 (Catapult S5, Melbourne, Australia) and six Physiolog 5 (GaitUp SA, Lausanne, Switzerland). Optimeye S5 comprised 3D accelerometer, a 3D gyroscope and 3D Magnetometer with sampling frequency 100 Hz. Physiolog 5 comprised of a 3D accelerometer, a 3D gyroscope and a barometric pressure sensor. The sampling frequency was set to 64 Hz for the barometric measurement, and 256 Hz for the accelerometer and gyroscope measurements. The IMUs were mounted on the upper back using an Optimeye S5 vest and with Velcro on the chest, lower back, left and right wrists, and in front of the binding on the left and right skis (Physiolog 5). 

After the test session, the IMU data were resampled to 100 Hz and synchronized in time with treadmill speed and incline. The Physilog 5 and the Optimeye S5 sensors were synchronized using 3 jumps, performed at the beginning and the end of the session.

### 2.3. Test Protocol

**Figure 2:**
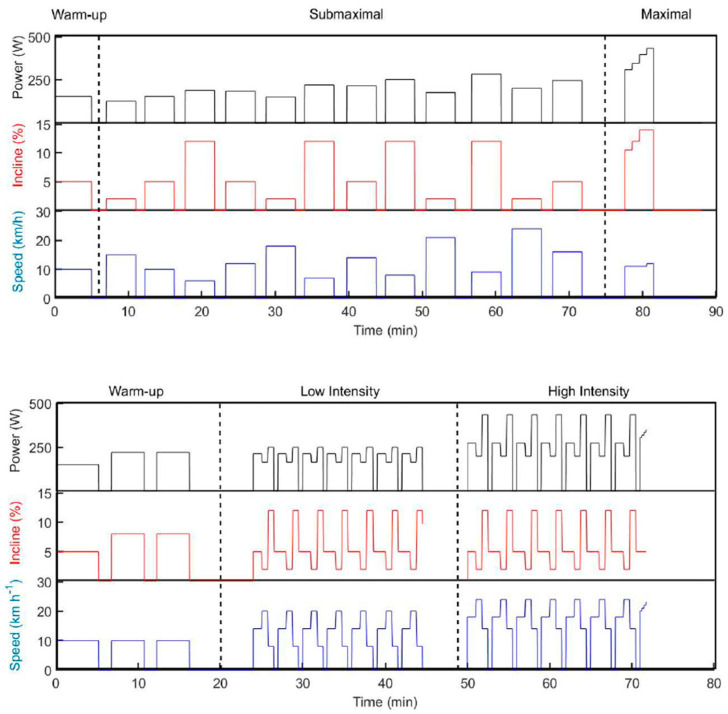
Top: Data from experiments at Day 1 showing submaximal bouts and a maximal incremental test. Bottom: Data from Day 2 showing 7 laps at low and high intensity.

For the participants, the protocol consisted of two consecutive testing days. Day 1 involved a 5 min warm-up, twelve submaximal exercise bouts of 4 min at constant speed, followed by a maximal incremental test. The twelve submaximal bouts consisted of three different sub-techniques (i.e., G2, G3 and G4) at four different intensities, performed in randomized order (G2: 12% incline at 6/7/8/9 km∙h^−1^, G3: 5% incline at 10/12/14/16 km∙h^−1^ and G4: 2% incline at 15/18/21/24 km∙h^−1^). A minimum of two minutes of recovery was given between each condition. The inclines and speeds employed represent typical inclines where these techniques are employed by elite skiers and were based on previous research. For the maximal incremental test, the starting incline and speed were 10.5% and 11 km∙h^−1^. The speed was then kept constant, while the incline was subsequently increased by 1.5% every minute until 14.0%. Thereafter, the speed was increased by 1 km∙h^−1^ every minute until exhaustion (Figure 2). 

Day 2 consisted of a 13 min warm-up before two 21 min stages of (a) low and (b) high (competition) intensity, with freely chosen technique across a simulated terrain profile on the treadmill. The track was organized as seven identical 3 min laps, consisting of four different segments that simulated a moderate uphill (5% incline), a flat segment (2% incline), a steep uphill (12% incline) and a simulated downhill (Figure 1). The profile of the track was designed according to standards of the International Ski Federation [30], where the standard sub-techniques could naturally be utilized. The high-intensity stage was immediately followed by an incremental all-out test at 5% incline, with gradually increasing speed (+1 km^−1^) every 15 s until exhaustion (Figure 2). 

### 2.4. Data Processing

For roller ski exercise on a treadmill, the work rate, equal to the average cycle propulsive power, can be calculated with high accuracy using the sum of power against gravity (*P_g_*) and friction (*P_f_*), where

(1) $$P_{g}=mg\sin\left(\alpha \right)v$$

(2) $$P_{f}=mg\cos\left(\alpha \right)\mu v$$

and where *m* is the mass of the skier, *g* the gravitational acceleration 9.81 m/s^2^, *α* the angle of treadmill incline, *v* the belt speed and *μ* the frictional coefficient.

The dataset used for machine learning consisted of the raw three-dimensional accelerometer and gyroscope data from a total of 7 IMU sensors (six Physiolog 5 and one Optimeye S5), the treadmill speed and incline, as well as the mass of the 13 athletes (except for the first experiment).

The data from different body sensors, mass and speed are represented by *U* as (3)–(6).

(3) $$B=G_{i}\left(A_{x}\right)||G_{i}\left(A_{y}\right)\left|\left|G_{i}\left(A_{z}\right)\right|\right|G_{i}\left(C_{x}\right)||G_{i}\left(C_{y}\right)||G_{i}{\left(C_{z}\right)}_{}{}_{}\;\;\;\;\;\;i=1,2,\ldots ,6$$

(4) $$D=T\left(A_{x}\right)||T\left(A_{y}\right)\left|\left|T\left(A_{z}\right)\right|\right|T\left(C_{x}\right)||T\left(C_{y}\right)||T{\left(C_{z}\right)}_{}{}_{}$$

(5) U=P||E||B||D

**Figure 3:**
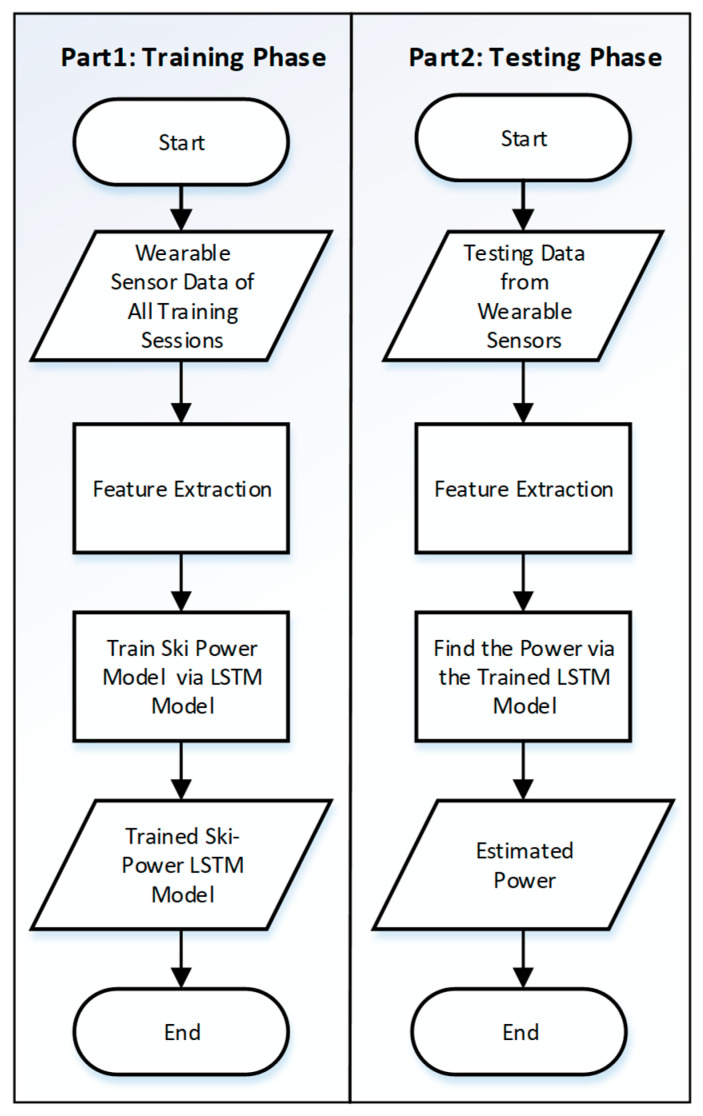
Flowchart of training and testing process of the proposed method.

In the above equations, *G* represents the IMU sensors, *A* accelerometer data along three axes, *C* gyroscope data along three axes, *T* catapult IMU sensor, *P* speed and *E* body masses of the athletes. Furthermore, the data were scaled through Gaussian standardization as (6).

(6) $$L=\frac{\left(U-µ\right)}{\sigma }$$

where *µ* and *σ* represents the mean and variance of the training dataset. *L* contains the standardized features used as inputs to the machine learning LSTM model. Figure 3 shows the flowchart of the proposed power estimation approach.

### 2.5. Machine Learning Model

**Figure 4:**
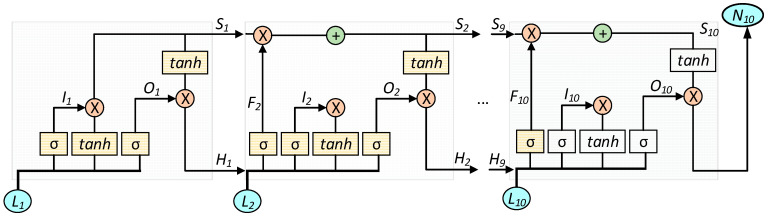
A basic structure of the LSTM-based RNN.

The recent success of machine learning models has been mostly made possible with the help of a combination of efficient deep machine learning algorithms applied on both visual and non-visual data in huge parametric space. Amongst different machine learning algorithms for sequential data modelling, LSTM overpowers others, such as the Artificial Neural Network (ANN) and CNN, which are mostly used for general and image data modelling, respectively. Hence, LSTM seems appropriate for this work, due to its time-sequence modelling capability. Figure 4 shows a sample RNN consisting of 10 LSTM units.

Each LSTM memory unit consists of three important gates: input, forget and the output gate. The input gate *I_t_* can be obtained as

(7) $$I_{t}=\sigma \left(W_{LI}L_{t}+W_{HI}H_{t-1}+{Ω}_{I}\right)$$

where *W* is a weight matrix, Ω bias and σ a sigmoid activation logistic function. The forget gate *F* can be obtained as

(8) $$F_{t}=\sigma \left(W_{LF}L_{t}+W_{HF}H_{t-1}+{Ω}_{F}\right)$$

The memory in the network can be stored in a state *S* that is expressed as

(9) $$S_{t}=F_{t}S_{t-1}+I_{t}\tanh(W_{LS}L_{t}+W_{HS}H_{t-1}+{Ω}_{S})$$

The output gate *O* determines what is going to be an output, as expressed by

(10) $$O_{t}=\sigma \left(W_{LO}L_{t}+W_{HO}H_{t-1}+{Ω}_{O}\right)$$

The hidden layer state *H* can be obtained as

(11) $$H_{t}=O_{t}\tanh(S_{t})$$

At last, the output *N* can be obtained as

(12) $$N=\mathrm{softmax}\left(W_{N}H_{l}+{Ω}_{N}\right)$$

where *l* represents the final LSTM unit in the network. 

In LSTM, the three gates (i.e., input, forget and output) manage the information flow in the network. The input gate *I* usually controls the ratio of the input as shown in (7). While calculating the memory state *S*, this input ratio has an effect on (9). The forget gate *F* basically decides to pass the previous memory from H_t−1_ using (8). Therefore, the ratio of previous memory is computed by (8) and used in (9). The output gate *O* takes the decision whether to pass the output of memory unit or not, as shown in (10). The hidden layer calculation is performed by (11), based on combining the results from output gate *O* and memory state *S*. Finally, a softmax function is usually used to model the final output as shown in (12). While training sequential data with an LSTM model, it makes the gradient exploding or vanishing with the help of these three gates. 

In this work, we adopt the LSTM model, having two LSTM hidden layers with 10 and 20 units, respectively, followed by a final output layer. The LSTM units and number of layers are chosen with empirical tests, starting with a limited number of units and increasing the number progressively, until the performance of the system stops improving during the experiments.

## 3. Experimental Setups and Results

**Figure 5:**
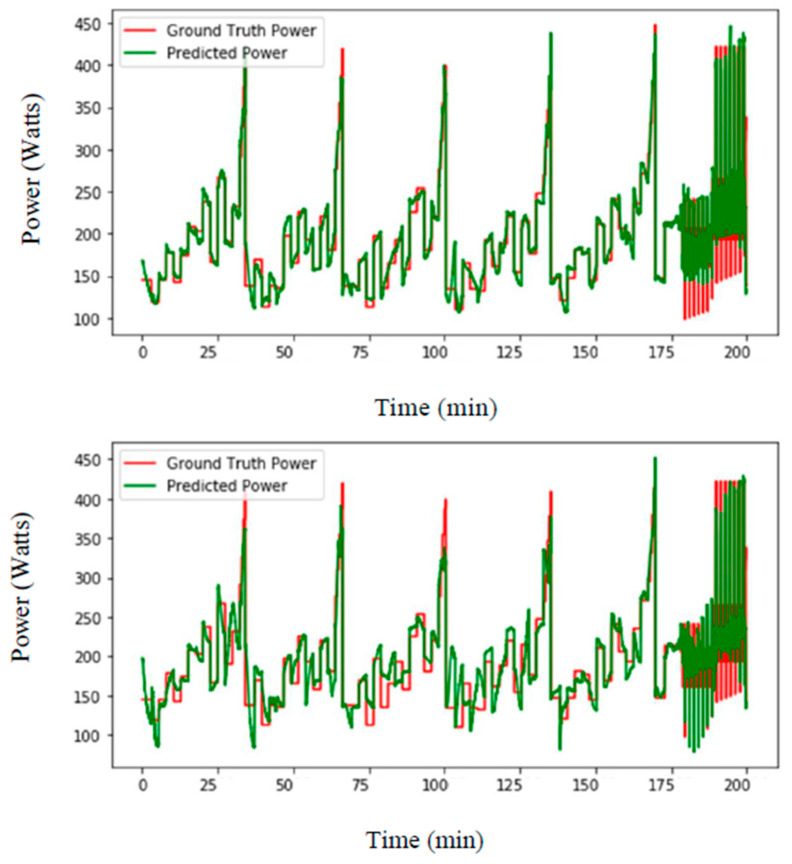
Top: LSTM-based prediction of ski power (watts) for training subjects 1–8 and testing subjects 9–13 where 5% (**top**) and 0.5% (**bottom**) of the data were removed from the testing subject to be used for training.

**Figure 6:**
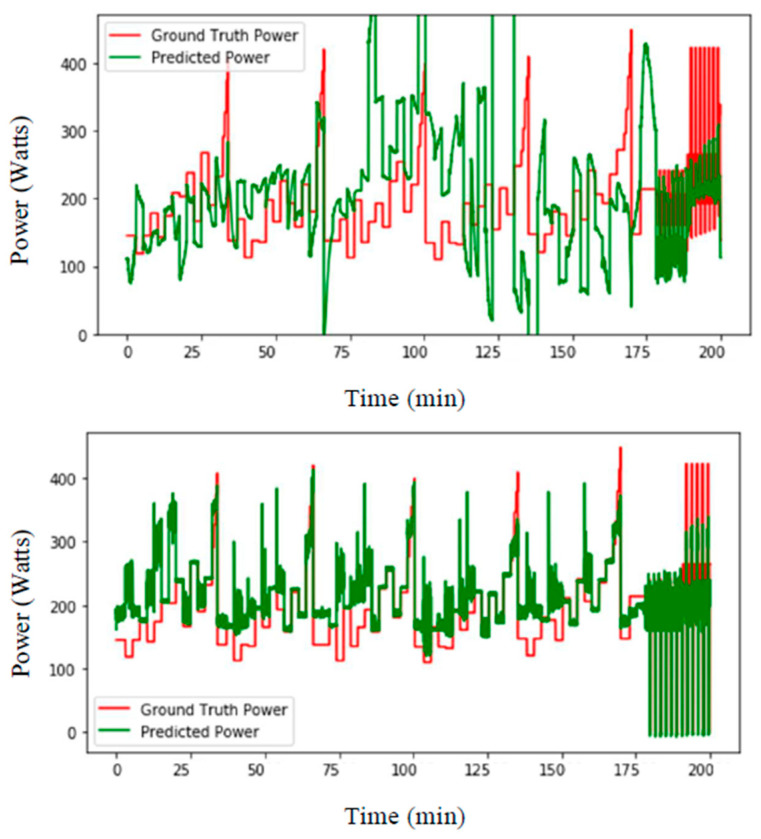
LSTM-based prediction of ski power (watts) with data from separate training and testing subjects (**top**) excluding and (**bottom**) including masses of the athletes where no data from the testing subjects were introduced in training.

In a first set of experiments, we split the dataset into two parts: eight subjects for training and five subjects for testing. The aim was to understand the influence of including data from the testing subjects in the training of LSTM-based neural networks. At first, 10% of random data from the testing subjects were included in the training data. Then, 5%, 1%, 0.5% and 0% of random data were also processed. Figure 5 shows the LSTM-based prediction of ski power, using training subjects 1–8 and testing subjects 9–13, where 5% and 0.5% data were removed from the testing subject to be used for training. The mean-squared prediction errors (in watt) and relative errors (in %) are reported in Table 1. The higher percentage of data from the testing subjects introduced for training provided the best performance. When no data from the testing subjects were introduced, the error was much higher than when even 0.5% of testing data were introduced. Based on these first results, the body masses of the athletes were provided as an input, and power prediction improved, as shown in Figure 6. The mean-squared prediction errors (watt) and relative errors (%), using the proposed approach, where 10%, 5%, 1%, 0.5% and 0% of the data that included subjects’ masses were taken away from the testing part to use for training, are also reported in Table 1. Introducing the athletes’ masses in the algorithm helped to improve the power prediction, with a particular efficiency when no data from the testing subjects was provided (i.e., decreasing from 50.9% to 17.9% error). It was then decided to perform a second experiment using a leave-one-subject-out method, to see if it was possible to obtain a better subject-independent model.

**Figure 7:**
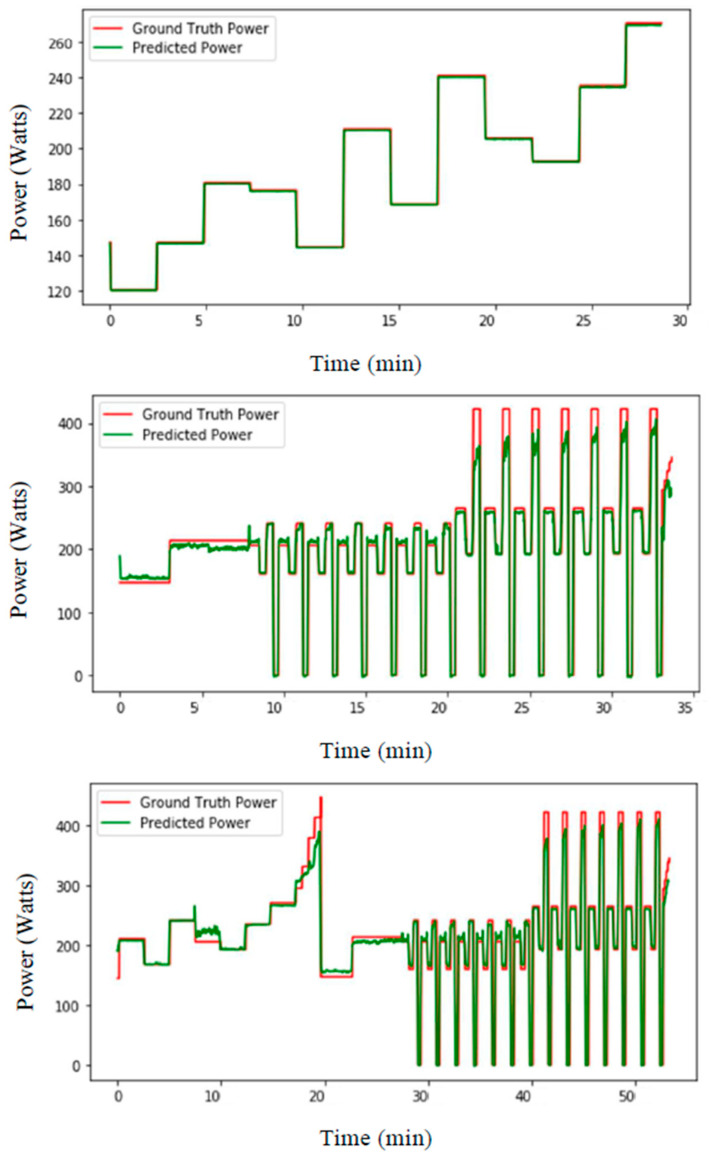
LSTM-based prediction of ski power (watts) from the data of (**top**) first, (**middle**) second and (**bottom**) both day(s) from subject 13 with training data of that day(s) from other subjects.

The second set of experiments started with training with the data of the first day of the protocol, and testing the data of that day. Then, the same experiments were carried out for the second day and finally for both days together. Figure 7 shows the prediction results for separate experiments of first, second and both day(s) for subject 13.

**Figure 8:**
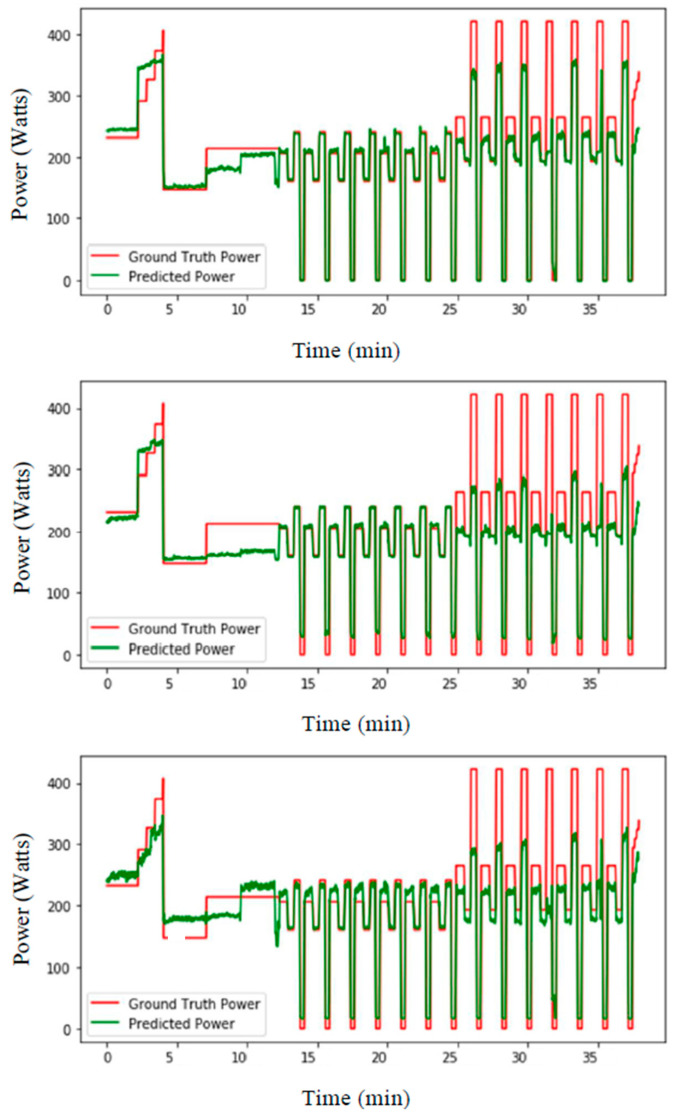
Long Short-Term Memory (**top**), typical large Artificial Neural Network (**middle**), and Convolutional Neural Network (**bottom**)-based prediction of ski power (watts) from the data of both days from subject 9, with training data of both days from other subjects.

**Figure 9:**
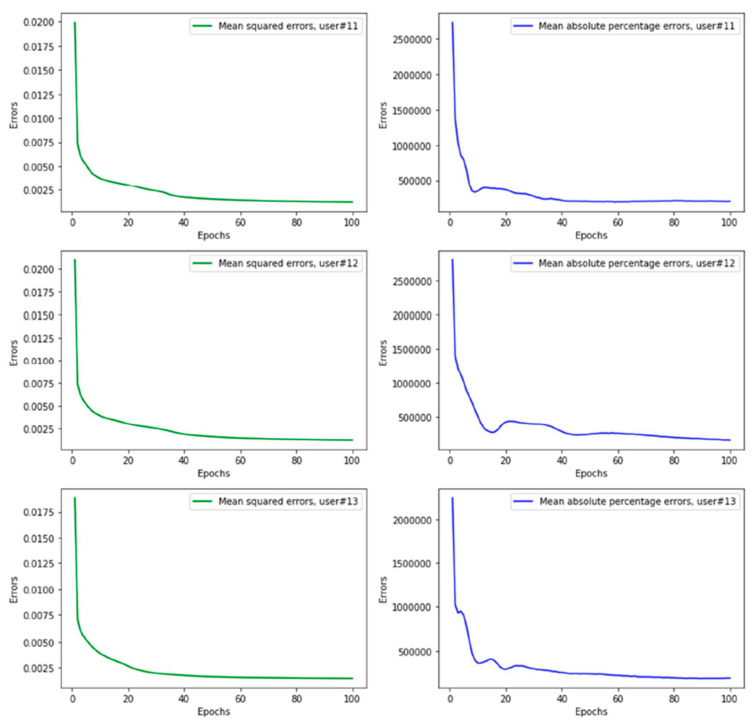
Mean-squared errors and mean absolute percentage errors with respect to 100 epochs from training models during the leave-one-subject-out method for cross-validation of subjects (**top**) 11, (**middle**) 12 and (**bottom**) 13.

The LSTM-based approach was then compared with other deep learning methods and showed better performance. Figure 8 shows the prediction results of the ski power using LSTM, ANN and CNN, respectively, for testing on subject 9, where the other subjects were used for training. Table 2 shows the mean-squared error for each subject, where the other subjects were used for training. Here, LSTM shows less errors compared to the other approaches. For the LSTM approach, the mean error for the estimated power is 42.1 ± 14.5 W, corresponding to a 11.6 ± 5.3% error. Figure 9 shows the mean-squared errors and mean absolute percentage errors for 100 epochs during the training of the LSTM model with the rest of the subjects and testing it for single subject (i.e., leave-one-subject-out method for cross-validation) for subjects 11, 12 and 13. The figure indicates that the errors smoothly decrease over time during the epochs, showing the robustness of the approach.

## 4. Discussion

The current study estimates mechanical power output by employing a time-sequential information-based deep Long Short-Term Memory (LSTM) neural network from multiple inertial measurement units (IMUs). By adopting two different setups of experiments, we yielded satisfactory accuracies from person-dependent power estimations (i.e., athlete-trained models), being within 3.5% error for 10% user-dependent data, while person-independent power estimation had an accuracy of less than 11.5% error. 

During the first experiments (i.e., person-dependent), where data did not include the body masses of the subjects and different subjects were used for training and testing the algorithm, the results show that the more of the user’s data were introduced in the training set, the higher the accuracy obtained. When there was no introduction of the testing user’s data in training at all, the relative error reached more than 50%. Thereafter, inclusion of the athletes’ body masses improved the accuracy for cases were there was no introduction of the testing user’s data in training. The positive effect of the inclusion of athletes’ body mass led to applying subject-independent power estimation in the second experiment, where we used the leave-one-subject-out method for cross-validation, confirming the generalization of the model to an independent data set [31].

In term of usability for performance and training analysis, an error of around 3.5%, as achieved by the individualized models, would give power prediction data applicable for research and for providing information to athletes and coaches at an elite level. However, applying this method would require each individual to go in the lab to record reference data. On the other hand, the user-independent model obtained using LSTM provided average results, with an 11.6% mean error. This accuracy may be high enough to provide recreational athletes with interesting and useful information to schedule and analyse their personal training, but would be too low to provide feedback for elite athletes and coaches when comparing performance in different conditions and across athletes. In addition, as the method has been developed with indoor data, generalisation for outdoor application, especially with skis on snow of changing conditions, would require further development and validation. Compared to the existing literature in roller skiing on the field, applying a model based on the power balance principle coupled with data from GNSS and IMUs [4], the current approach shows potential to improve power prediction accuracy, especially for the individualized models and at high skiing speeds. It was found that the estimated propulsive power for an outdoor roller ski was most accurate at low skiing speeds with an uncertainty of 0.09 W kg^−1^ (around 7 W), similar to the person-dependent model in the current study, with accuracy decreasing to 0.58 W kg^−1^ (around 46 W) at high skiing speeds, similar to the person-independent model in the current study. 

Regarding the accuracy obtained by machine learning models, LSTM network models displayed slightly better results than CNN and ANN models, with lower mean- squared errors across experiments, confirming their better suitability to learn and remember over long sequences of input data where spatial correlations are not of interest. Even though it can be argued that CNNs are faster by design, due to the computations in CNNs occurring in parallel, while LSTMs need to be processed sequentially, CNNs’ rigid, forward structure limits their applicability for time sequence problems. Additionally, LSTM models are optimized for learning from raw time series data directly, and in turn do not require the time and expertise to engineer input features. Hence, this approach is far more widely applicable and opens new possibilities for the study of athletic performance from wearable sensor data. However, further tuning of hyperparameters of CNN models may produce similar results, such as the proposed LSTM-based one.

## 5. Conclusions

In this study, we developed a multimodal power estimation model for roller ski skating on a treadmill, using an LSTM deep learning method on data from multiple body-worn IMUs. Overall, the user-dependent model allows for precise (3.5% error) estimation of roller skiing power output, with the limitation that some training data must be provided for the athlete to achieve this accuracy. The user-independent model provides less accurate estimation (11.5% error). However, the accuracy may be sufficient for providing valuable information for recreational skiers and there is no need to record training data for each athlete in the laboratory. Finally, LSTM networks performed better then CNN and ANN networks, confirming their suitability for time-sequential data.

## Figures and Tables

**Table 1:** Mean-squared errors (MSE) in watts (W) and relative error (RE) in % of the estimated power for partially user-dependent approach using Long Short-Term Memory LSTM with or without body mass included in the data.

User-Dependent Data Included in Training	Body Mass not Included		Body Mass Included	
	MSE (W)	RE (%)	MSE (W)	RE (%)
10%	11.5	3.8	10.9	3.5
5%	14.1	5.0	13.7	4.9
1%	27.0	10.0	24.6	8.9
0.5%	36.5	14.3	34.1	12.9
0%	144.4	50.9	57.2	17.9

**Table 2:** Mean-squared errors (MSE) in watts (W) and relative error (RE) in % for each subject where the other subjects were used for training, using Long Short-Term Memory (LSTM), Convolutional Neural Network (CNN) and Artificial Neural Network (ANN).

				LSTM		CNN		ANN	
	Age (year)	Height (cm)	Mass (kg)	MSE (W)	RE (%)	MSE (W)	RE (%)	MSE (W)	RE (%)
Subject 1	28	186.5	83.1	35.8	9.4	49.9	13.1	49.3	12.9
Subject 2	21	180	73.1	54.0	14.3	64.3	17.0	62.3	16.5
Subject 3	25	194.5	84.6	58.0	12.6	62.9	13.6	62.9	13.6
Subject 4	24	190.5	81.1	55.9	12.5	61.2	13.7	62.0	13.9
Subject 5	29	181	78.5	49.6	17.4	49.7	17.4	50.3	17.6
Subject 6	28	185	77.5	48.6	18.5	56.8	21.6	51.5	19.6
Subject 7	22	180.1	83.5	56.4	18.5	58.5	19.2	57.6	18.9
Subject 8	27	196.5	91.6	57.6	16.7	60.4	17.5	60.9	17.6
Subject 9	26	180.5	78.1	20.3	4.7	62.9	14.5	61.9	14.3
Subject 10	21	181	74.1	26.2	5.3	30.2	6.1	27.4	5.5
Subject 11	23	183.5	74	21.7	4.5	24.9	5.1	24.8	5.1
Subject 12	22	176.5	72.1	33.1	8.5	33.6	8.7	34.0	8.8
Subject 13	26	177	79.3	30.3	7.5	31.7	7.9	33.9	8.4
Mean	24.8	184.0	79.28	42.1	11.6	49.8	13.5	49.1	13.3
SD	2.8	6.3	5.52	14.5	5.3	14.5	5.2	14.2	4.9

Mean values and standard deviation (SD) are presented.

## Data Availability

The data presented in this study are available on request from the corresponding author. The data are not publicly available due to privacy restrictions.
